# Exploring the Microalga *Euglena cantabrica* by Pressurized Liquid Extraction to Obtain Bioactive Compounds

**DOI:** 10.3390/md18060308

**Published:** 2020-06-12

**Authors:** Nerea Muñóz-Almagro, Bienvenida Gilbert-López, Pozuelo-Rollón M. Carmen, Yolanda García-Fernandez, Carlos Almeida, Mar Villamiel, Jose A. Mendiola, Elena Ibáñez

**Affiliations:** 1 Institute of Research in Food Sciences, CIAL (UAM-CSIC), Nicolás Cabrera 9, Campus Universitario de Cantoblanco. 28049 Madrid, Spain; nerea.almagro@csic.es (N.M.-A.); mcpozuelorollon@gmail.com (P.-R.M.C.); m.villamiel@csic.es (M.V.); elena.ibanez@csic.es (E.I.); 2 Analytical Chemistry Research Group (FQM-323), Department of Physical and Analytical Chemistry, University of Jaén, Campus Las Lagunillas edif. B3, 23071 Jaén, Spain; bgilbert@ujaen.es; 3 Spanish Bank of algae (BEA), Instituto de Oceanografía y Cambio Global (IOCAG), Fundación Canaria Parque Científico y Tecnológico, Universidad de Las Palmas de Gran Canaria (ULPG), 35230 Las Palmas, Canary Islands, Spain; garcia.fernandez.yolanda@gmail.com (Y.G.-F.); calmeida@marinebiotechnology.org (C.A.)

**Keywords:** *Euglena cantabrica*, pressurized liquid extraction, carotenoid, carbohydrates, response surface, microalga, paramylon

## Abstract

In the present study, the chemical composition of the microalga *Euglena cantabrica* was investigated. The extraction of bioactive compounds was done using pressurized liquid extraction (PLE) at different temperatures (40–180 °C) and using green solvents (ethanol-water mixtures). A statistical design of experiments was used to optimize the maximum antioxidant capacity of the extracts by response surface methodology. The antioxidant capacity was determined through the inhibition of 2,2’-azino-bis-3-ethylbenzothiazoline-6-sulfonic acid (ABTS) and 1,1-diphenyl-2-picrylhydrazyl (DPPH) radicals, while the chemical analyses of the extracts were carried out using different chromatographic techniques. Chlorophylls and carotenoids were analyzed by high-performance liquid chromatography coupled to a diode array detector and mass spectrometry (HPLC-DAD-MS/MS) and carbohydrates by gas chromatography with flame ionization detection (GC-FID) and high-pressure size-exclusion chromatography coupled to an evaporative light-scattering detector (HPSEC-ELSD). The results showed different possibilities for the extraction conditions, depending on the desired bioactivity or chemical composition. Briefly, (i) mixtures of ethanol-water containing around 40% ethanol at 180 °C gave the best antioxidant capacity, (ii) mixtures containing around 50% ethanol at 110 °C gave the best yield of β-glucan paramylon, and (iii) the use of pure ethanol at a low temperature (40 °C) is the best choice for the recovery of carotenoids such as diatoxanthin. Summing up, *E. cantabrica* seems to be a good candidate to be used in biorefinery to obtain different bioactive compounds.

## 1. Introduction

In spite of the fact that macroalgae have been used in China since 1000 b.c. and for ethnical uses in the Mayan culture and certain African tribes, they have not been used in a massive way until the middle of the 20th century. The way to cultivate them was investigated and simplified at the beginning of the 20th century [1]. Among others, the most important microalgal metabolites are carbohydrates, proteins, lipids, carotenoids, phenolic compounds, and phytohormones with a plethora of bioactive properties that can promote good health (bioactive compounds) [2]. Therefore, microalgae have a huge potential, because they are a vast, unexploited reservoir of new compounds that are waiting to be discovered. In fact, according 2017 estimations, a total of US $6.5 billion is the global market value of microalgae, being US $2.5 billion in the bioactive food ingredients category [3]. Among the different species, *Euglena* spp. are considered as an adequate medium for the large-scale production of metabolites without risk to humans. This is due to the extraordinary metabolic ability even comparable with multicellular organisms. It is known that *Euglena* spp. contain approximately 34% of carbohydrates, paramylon being the most important regarding its bioactivity and amount, up to 70–80% [4]. Among the β-glucans, paramylon is composed of only β (1,3) bonds. Further, paramylon exists in a granular form in Euglena cells of all of species and varieties, and the number, the shape, and the uniformity of the particles of paramylon are characterized depending on the species. As it is the case with the other β-glucans, paramylon is expected to have functionality, but much remains unknown regarding the mechanism of action thereof. Its unique structural features provide techno-functional properties and modulation of the immune system, decrease of cholesterol, and control the postprandial answer of humans to glucose [5]. 

**Figure 1:**
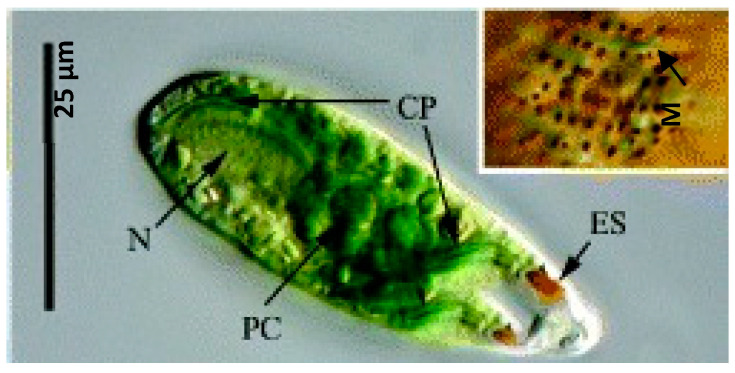
Microscopic view of *Euglena cantabrica*. N (nucleus), CP (chloroplast), ES (eyespot), PC (paramylon center), and M (mucocysts). Image from Shin and Triemer, 2004 [6] with permission of John Wiley and Sons, Ltd.

*Euglena cantabrica* is a green unicellular microalga belonging to the genus *Euglena*, which results from the combination of the Greek words “eu” (good) and “glene” (eyeball) and refers to the distinct eyespot that can be seen in most euglenoid cells [5]. The eyespot, also called stigma, is a photoreceptive pigment located near to the flagella and involved in the movement of the microalga in response to light intensity (phototaxis) [5]. The information about *E. cantabrica* available in the literature is limited and mainly related to taxonomical classifications [6,7,8]. Figure 1 shows the main morphological characteristics of *E. cantabrica*, which include the presence of spherical mucocysts, a stellate chloroplast, and a paramylon center, in addition to the eyespot and the flagella [6,8].

Regarding the composition of *E. cantabrica*, the β-1,3-glucan called paramylon is the principal reserve carbohydrate—as it is common within the genus *Euglena*—and it is located in structures called paramylon centers [8]. Other species from the genus *Euglena*, such as *Euglena gracilis*, have been cultivated at a large scale for the production of paramylon [9]. Nevertheless, there is no information about the recovery of paramylon or other carbohydrates from *E. cantabrica*. Additionally, the pigment’s composition of *E. cantabrica* has not been studied, while the presence of astaxanthin, β-carotene, or diatoxanthin have been reported in different species of Euglena [10,11]. To the best of our knowledge, there has been published only a pioneer paper by Jerez-Martel et al. in 2017 [12] that shows the first results about the phenol compositions of *E. cantabrica* and the antioxidant capacity of these extracts. Conventional extraction with pure water or pure methanol was assayed in that work [12]. 

Recent studies have demonstrated the ability of pressurized techniques, such as pressurized liquid extraction (PLE) or supercritical fluid extraction (SFE) combined with green solvents—such as water, ethanol, or carbon dioxide, among others—to extract bioactive compounds from algae [2,13]. Among the advantages of PLE versus conventional extraction techniques, we can find the short extraction times, low volume used, and the possibility of extracting thermally labile compounds at high temperatures without degradation. Moreover, we can find a wide range of solvents that can be used, especially green solvents. The high pressure employed in these techniques allows the solvent to be in a liquid state above its boiling point, offering unique properties to extract valuable compounds from complex natural matrices [14]. For instance, PLE has been employed for the extraction of antioxidants from *Dunaliella salina* [15] and for the extraction of pigments from *Neochloris oleoabundans* [16] or *Phaeodactylum tricornutum* [17] or even carbohydrates and amino acids [18] and lipids [19]. Finally, the combination of several pressurized techniques has been revealed as a good approach for the fractionation of microalgae in a biorefinery approach [20,21]. Therefore, the objective of this study was to give an insight into the chemical composition of *Euglena cantabrica* in order to evaluate its potential as a source of bioactive products obtained using green technologies. 

## 2. Results and Discussion

### 2.1. Design of Experiments (DoE) for Pressurized Liquid Extraction (PLE)

To optimize the extraction conditions to obtain bioactives from *E. cantabrica* by PLE, a design of experiments to study the interaction of two factors was selected. A factorial design type 32 (two factors at three conditions or levels) with three additional replicates in the central point was used in order to study the influence of the extraction temperatures (40 °C, 110 °C, and 180 °C) and solvent compositions (0% ethanol (water), 50%, and 100%) in the response variables. This means that 12 assays were done: nine of the factorial design and three additional replicates of the central point (110 °C, 50% ethanol) in order to establish the error. Those solvents were selected in order to obtain as much paramylon and carotenoids as possible, covering the widest range of temperatures possible to increase the yield. The selected response variables were the extraction yield (mass of extract per mass of dry weight, expressed in percentage), total phenols content (TPC), Trolox equivalent antioxidant capacity (TEAC) obtained by the ABTS (2,2’-azino-bis(3-ethylbenzothiazoline-6-sulfonic acid) diammonium salt) assay, and EC_50_ (effective concentration of extract able to decrease the concentration of a radical to the half) obtained by DPPH (1,1-diphenyl-2-picrylhydrazyl) assay. The results obtained for all the responses variables are displayed in Table 1.

The quadratic model proposed for each response variable (Yi) was:

Yi = β_0_ + β_1_·A + β_2_·S + β_1,1_·A^2^ + β_1,2_·A·B + β_2,2_·B^2^ + ε,

where A is the temperature, B is the % EtOH (solvent composition), β_0_ is the intercept, β_1_ and β_2_ are the linear coefficients, β_1,1_ and β_2,2_ are the quadratic coefficients, β_1,2_ is the interaction coefficient, and ε is the error variable.

**Figure 2:**
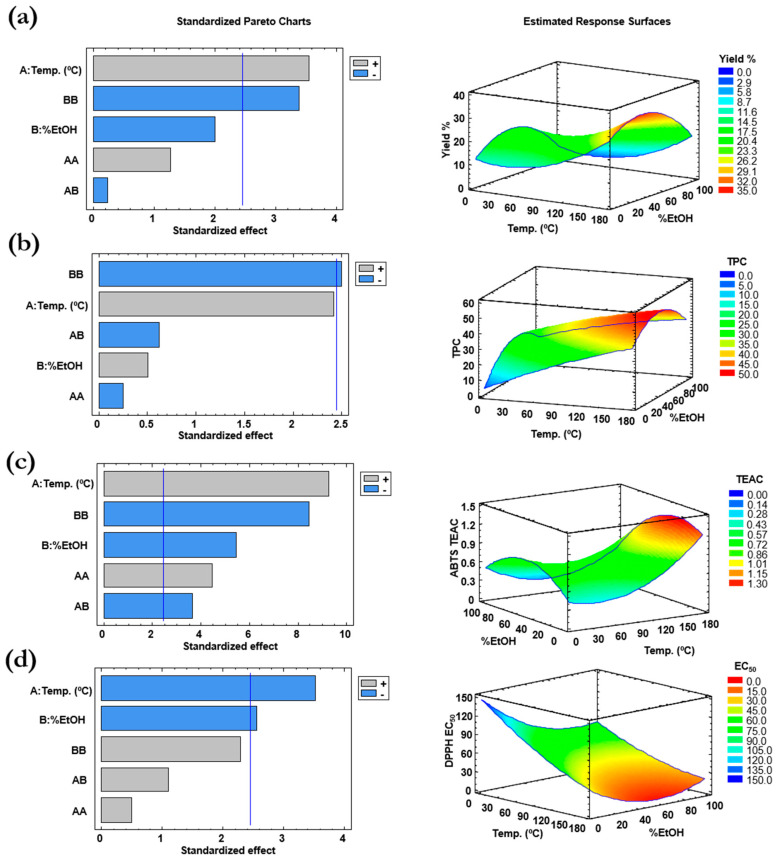
Pareto’s diagrams and estimated response surfaces for (**a**) extraction yield, (**b**) total phenols, and antioxidant capacity measured by (**c**) ABTS test and (**d**) DPPH test. Vertical line of Pareto’s diagrams shows a confidence range of 95%, and the color shows if the influence of the factors is positive (increasing the value of the response variable, grey color) or negative (blue color).

An ANOVA (analysis of variance) test was performed for each response variable, and the model adequacy was evaluated by the determination coefficient (R^2^) and the residual standard deviation (RSD) at a confidence level of 95% (*p* = 0.05). Response surface plots were developed using the fitted quadratic polynomial obtained, and the software from the fitted model provided the optimum conditions. Pareto’s diagrams, which point out the coefficients of the model that affect significantly the results together with the response surfaces, are shown in Figure 2. Following, the effects of the temperature and solvent composition on each of the response variables is discussed.

Extraction yield. As it is shown in Figure 2a, the best yields are obtained using a solvent composition around 50% of ethanol. This may be due to the polarity of the extracted compounds, which seems to be in between water and ethanol. In addition, the extraction temperature increases the yield as a result of the increase of the compounds’ solubility and decrease in solvent viscosity due to temperature; thus, an increase of the transfer ratio of the sample matter to the solvent is produced. Similar results have been reported previously for other microalgae [17,21]. In this case, the Pareto’s diagram shows that the temperature gives the most significant influence on the extraction. The ANOVA test showed a R^2^ = 0.8245, which means that the model is able to explain 82.45% of the variability in yield, and, using this fitted model, the optimum conditions provided by the statistical software Statgraphics Centurion (version XVI) are 180 °C and 38.7% of ethanol. The maximum yield predicted is 34.2%, which is between the yields reported by Jerez-Martel et al. [12] using water (25.7% yield) and methanol (57.9% yield) for 40 min of conventional extraction at room temperature.

Total phenols content (TPC). The TPC was measured by means of the Folin-Ciocalteau test, and the results were expressed as milligrams of gallic acid equivalents per gram of extract (mg GAE/g extract). The response surface depicted in Figure 2b shows a similar trend to the extraction yield, which means that the total phenols content increases with the temperature and with an intermediate percentage of ethanol. According to the lack-of-fit test, since the *p*-value obtained (0.22) is greater than 0.05, the model appears to be adequate for the observed data at the 95.0% confidence level. However, the model is able to explain only 69.99% (R^2^ = 0.69993) of the variability in the TPC and it does not explain the close high values of the TPC obtained in the experiments using 110 °C, 50% EtOH and using 180 °C, 0% EtOH (see Table 1). The mathematical model proposes that the best extraction conditions to get the highest phenols content is obtained with the highest temperature (180 °C) using 48.4% of ethanol. 

Antioxidant capacity. The antioxidant capacity of the extracts is usually determined by in vitro colorimetric assays, and those with higher antioxidant capacities are then selected for further bioactivity assays. In the present work, the antioxidant capacity of the extracts has been tested by two assays based on the ability of the extracts to reduce the concentration of different radicals, namely ABTS and DPPH. In general terms, it can be stated that more polar compounds inhibit more efficiently the ABTS radical, while less polar compounds inhibit more efficiently the DPPH radical, so different results may be observed in these assays, because they are driven by different reaction mechanisms.

The results obtained from the ABTS assay are expressed as TEAC—that is, mmol of Trolox equivalents (TE) per gram of extract, since Trolox is used as the analytical standard. According to the Pareto’s chart depicted in Figure 2c, all the coefficients of the model significantly affects the results of the TEAC values, although the most important effect is related to the temperature. The response surface is similar to those obtained for the yield and TPC variables, showing an increase of the antioxidant capacity against the ABTS radical at higher temperatures and a solvent composition close to 50% EtOH. The R^2^ coefficient indicates that the model as fitted explains 97.13% of the variability in TEAC, providing as the theoretical optimum extraction conditions a temperature of 180 °C and a solvent composition of 30.4% of ethanol.

On the other hand, the results obtained from the DPPH scavenging test are expressed as the effective concentration of the extract (in µg/mL) able to decrease the radical concentration by 50% (EC_50_). Therefore, the higher the EC_50_, the lower the antioxidant capacity, since it needs a higher concentration of extract to scavenge the 50% of the DPPH radical concentration. That means that the optimization of this response variable consists of minimizing its value. The Pareto’s diagram depicted in Figure 2d shows that the temperature has the most important influence on the extraction, followed by the percentage of ethanol in the solvent. The R^2^ coefficient obtained in the ANOVA test indicates that the model as fitted explains 81.86% of the variability of the results. Since higher temperatures and higher percentages of ethanol decrease the EC_50_ value, it is not surprising that the extraction conditions proposed by the model to get the best antioxidant capacity are 180 °C and 58.8% ethanol.

At this point, it is interesting to remark that the antioxidant capacity found in our PLE extracts measured by DPPH is 100-fold higher than that found by conventional solid-liquid extraction [12], despite that their yields were higher. In the work by Jerez-Martel et al. [12], using 1 mg/mL, authors obtained an inhibition of 48% with water and 71% with methanol, both at room temperature. Comparing with our best extract at 180 °C, 100% ethanol, the inhibition found was 50% (EC_50_) using 7.5 µg/mL (0.0075 mg/mL using their units).

### 2.2. Chemical Characterization

The chemical characterization of phenols present in *E. cantabrica* has been recently studied [12]; authors reported the presence of gallic acid, protocatechuic acid, (+)-catechin, chlorogenic acid, and (−)-epicatechin. In order to broaden this information, other groups of metabolites have been targeted in the present work. Different chromatographic techniques have been employed for the identification of carotenoids, chlorophylls, and carbohydrates present in the different PLE extracts. A detailed study of the lipid composition was discarded, since the total amount of lipids found in all PLE extracts of *E. cantabrica* was below 0.1%. 

The total lipid content of the extracts was calculated as the dry weight of the material soluble in CHCl_3_:MeOH (2:1) from the dry PLE extract expressed as a percentage. The results showed that the lipids content is higher at higher temperatures and with high amounts of ethanol in the extraction solvent, as expected according to solvent polarity. However, in any case, the amount of lipids is not relevant (below 0.1% lipids in the extract).

#### 2.2.1. Analysis of Pigments by Liquid Chromatography-diode Array Detection Followed by Atmospheric Pressure Chemical Ionization Tandem Mass Spectrometry (HPLC-DAD-APCI-MS/MS)

**Figure 3:**
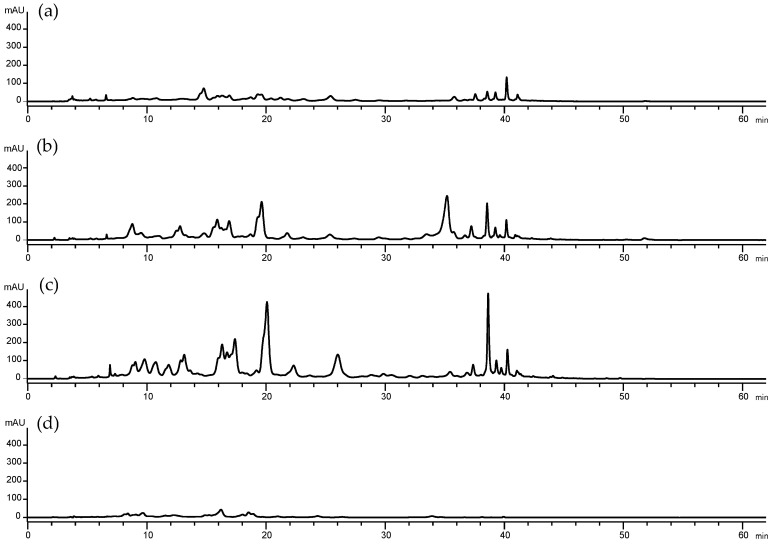
High-performance liquid chromatography coupled to a diode array detector (HPLC-DAD) chromatographic profile obtained at 450 nm from the PLE extracts carried out at (**a**) 180 °C, 100% EtOH (solvent composition); (**b**) 110 °C, 100% EtOH; (**c**) 40 °C, 100% EtOH; and (**d**) 110°C, 50% EtOH.

**Figure 4:**
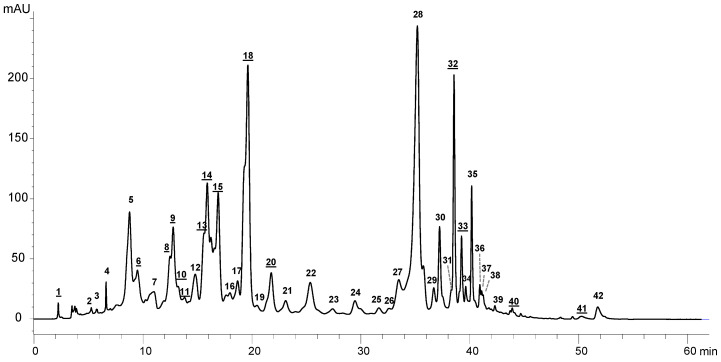
HPLC-DAD profile corresponding to the analysis of pigments obtained at 450 nm from an extract done at 110 °C and 100% ethanol. Carotenoids are represented with an underlined number and chlorophylls without underline.

The main pigments present in *E. cantabrica* are carotenoids (present in the photoreceptive stigma or eye spot) and chlorophylls (the photosynthetic pigments located in the chloroplasts). In order to determine the photosynthetic pigments present in the different PLE extracts, they were analyzed by HPLC-DAD-APCI-MS/MS. A tentative identification of the pigments present in the extracts of *E. cantabrica* has been performed for the first time considering their retention time (Rt), the UV-Vis absorbance spectra, the mass spectrometry results, and the data available in the literature [11,22,23,24,25]. Due to their hydrophobicity, carotenoids and chlorophylls are more efficiently extracted with pure ethanol, as can be observed in Figure 3. Chromatographic peaks absorbing at 450 nm are significantly less intense using 50% EtOH (Figure 3d) than using 100% EtOH (Figure 3a–c). It can be also observed that there is a strong influence of the temperature that can cause the degradation of carotenoids pigments, as it has been suggested in other studies [14,15,16,17]. This fact can explain the different profiles shown in Figure 3. Comparing the peak abundance obtained at 180 °C with 100% EtOH (Figure 3a) with 100% EtOH at 110 °C (Figure 3b) and at 40 °C (Figure 3b,c, respectively), lower amounts of peaks are obtained. Moreover, the chromatogram obtained with 100% EtOH at 110 °C (Figure 3b), shows an intense peak at 35.2 min (Figure 4, peak 28) that is not present in the other chromatograms, even though the chromatogram obtained at 40 °C is more intense (Figure 3c). For these reasons, the extract obtained at 110 °C has been selected to illustrate the identification of the pigments extracted by PLE from *E. cantabrica* (Figure 4).

The chromatogram corresponding to the sample extracted at 110 °C with 100% ethanol is shown in Figure 4. The main peak observed with a spectrum typical of carotenoids is peak number 18 (Rt = 19.6 min), which has been tentatively identified as diatoxanthin. On the other hand, the main peak observed with a spectrum typical of chlorophylls is peak number 28 (Rt = 35.2 min), which has been identified as a pheophytin b. This is the main peak observed at 110 °C (Figure 3b and Figure 4), and it is neither present at 40 °C (Figure 3c) nor at 180 °C (Figure 3a). Peak number 32 is one of the main peaks present in the extracts obtained at 40 °C and at 110 °C and corresponds to a carotenoid that has not been identified due to an inefficient ionization.

The identification of all the pigments present in the PLE extract obtained at 110 °C with 100% ethanol is detailed in Table 2. In total, twenty-four chlorophyll-related compounds have been detected, together with fifteen carotenoids (peaks with underlined numbers) and three unidentified mixtures of compounds (peaks 17, 36, and 42). As aforementioned, the main carotenoid present in the extract was diatoxanthin (peak 18), which has been tentatively identified according to its absorbance spectrum, its protonated molecule (*m/z* 567.5), and one characteristic fragment corresponding to the dehydrated molecule (*m/z* 549.7). The retention time matched with the one observed in *Phaeodactylum tricornutum* using a similar chromatographic method [17]. Diatoxanthin has been reported as a main carotenoid in microalgae from the *Euglenophyta* division [26] and has been previously identified in another species of the genus *Euglena*, namely *E. sanguinea* [11].

Several derivatives of both chlorophylls *a* and b have been tentatively identified according to their UV-Vis and MS spectral data [23,24,25], being the most intense peak of this group the one corresponding to pheophytin b (Figure 4 and Table 2, peak 28). Pheophytin b results when the Mg atom is removed from the tetrapyrrole ring of chlorophyll b. It has been identified from the absorbance maxima, its protonated molecule (*m/z* 885.8) and two characteristic fragments resulting from the loss of the phytyl group (*m/z* 607.4) and the subsequent fragmentation of the propionic chain (*m/z* 547.3). Similarly, peak 27—which is not the baseline separated from peak 28—has been assigned to pheophytin b’. Peaks 29 and 30 have been identified as pheophytin *a* and *a*’, showing the protonated molecule (*m/z* 871.9) and the same fragmentation scheme described for pheophytin b. The compound resulting from the de-esterification of the phytyl chain of pheophytin *a* is called pheophorbide *a* (*m/z* 593.5), which has been also detected in the extract (peak 7). On the contrary, pheophorbide b has not been found.

Other derivatives called pyropheophytins are formed through the decarboxylation of pheophytins and have been also identified in the PLE extract of *E. cantabrica*. Peak 35 has been assigned to pyropheophytin b (*m/z* 827.5), while peaks 37 and 38 have been identified as pyropheophytin *a* and *a’* (*m/z* 813.9), considering their protonated molecules and their respective fragments corresponding to the loss of the phytyl group. Pyropheophorbides are formed through the de-esterification of the phytyl chain of pyropheophytins, and both pyropheophorbide b (peak 12, *m/z* 549.4) and pyropheophorbide *a* (peak 16, *m/z* 535.5) have been identified in the extract. Pyropheophorbide b show a fragment of *m/z* 521.5 that correspond to CO fragmentation, which has been reported as exclusive of chlorophyll b derivatives [24,25].

It is important to note that chlorophylls *a* and b are located in the chloroplasts, and the development of chloroplasts in the *Euglena* genus is influenced by the light cycle. In addition, diatoxanthin has been associated with the photoprotection of *E. gracilis* during the long-term acclimation to light-induced stress [27]. Therefore, the profile of pigments in *E. cantabrica* depends on both the cultivation and the extraction conditions.

#### 2.2.2. Characterization of Carbohydrates 

**Figure 5:**
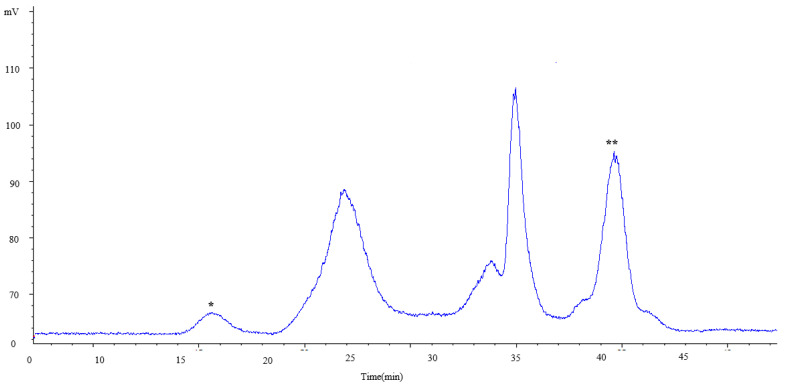
HPSEC-ELSD profile of the carbohydrate fraction corresponding to bioactive component extractions at 110 °C with 50% of EtOH. Peaks labeled as * and ** were out of the range of estimation of the method.

**Figure 6:**
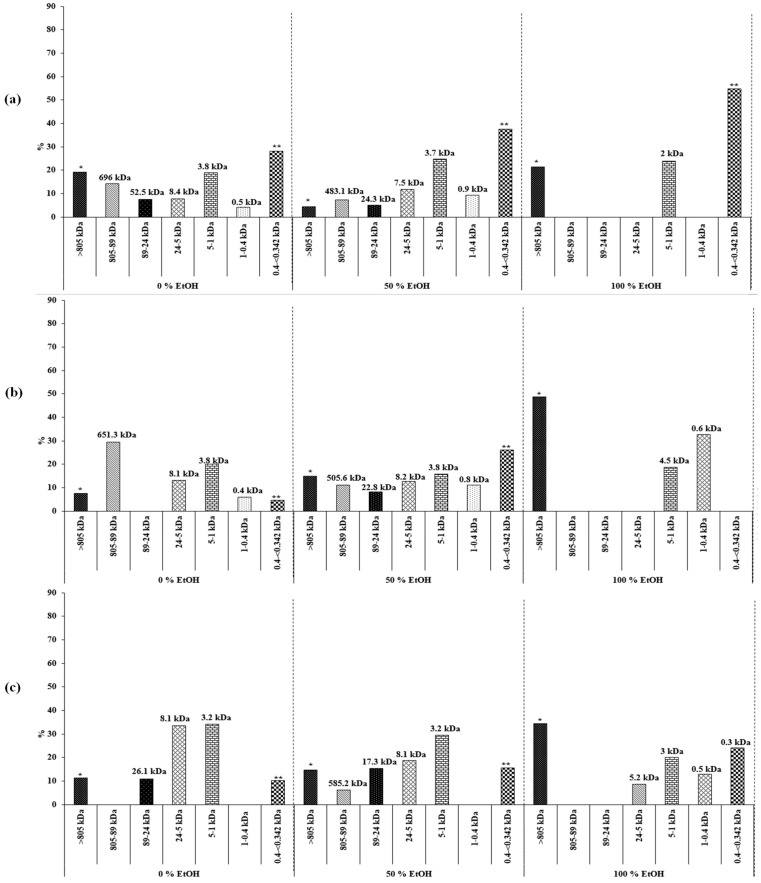
Distribution and abundance of the different carbohydrate fractions according to the estimations of the molecular weight (Mw) obtained by HPSEC-ELSD of the extractions carried out at 40 (**a**), 110 (**b**), and 180 °C (**c**).

In order to estimate the molecular weight (Mw) of the carbohydrates present in the samples, a HPSEC-ELSD (high-pressure size-exclusion chromatography coupled to an evaporative light-scattering detector) analysis was carried out. The chromatographic profiles were different depending on the extraction conditions used, since the number of peaks varied from two to eight. Figure 5 shows, as an example, the profile corresponding to a sample subjected to an extraction at 110 °C with 50% of EtOH. As can be seen, several of the observed peaks, the first (labeled in Figure 5 as *) and the latter (labeled as **), were out of the range of estimation of the method, in agreement with conditions indicated in the Materials and Methods section. The peak with a maximum of 25 min corresponded to an average Mw of 506 kDa. Previous studies have reported that other species of Euglena (*E. gracilis*) possesses a β-glucan (paramylon) with a Mw of 500 kDa [28]. Therefore, it is plausible that this peak was due to paramylon. The other peaks could correspond to glycans different from paramylon with lower Mw. Although in *E. gracilis*, the major carbohydrate is paramylon, other more structurally complex polymers are also present [28]. Figure 6 provides detailed information about the distribution and abundance (under the chromatographic conditions here used) of the different carbohydrate fractions at different PLE conditions, according to the estimation of the Mw obtained by HPSEC-ELSD.

Regarding the distribution of the different fractions of carbohydrates found in the extractions (Figure 6), the use of 100% EtOH is inadequate for the extraction of this polysaccharide, regardless of the temperature. At 0% of EtOH, a peak with a Mw higher than paramylon is observed at 40 °C (696 kDa) and 110 °C (651.3 kDa). Considering the extractions with 50% ethanol, together with paramylon (483.1–585.2 kDa), up to four molecular species with Mw lower than 17 kDa were observed, probably glycans that can be observed in microalgae from the same gender [28]. It is clear that temperatures of 40 and 180 °C led to higher proportions of these poly- or oligosaccharides. Therefore, it seems that the best extraction conditions for obtaining this β-glucan could be 110 °C and 50% of EtOH. As indicated above, the presence of this polysaccharide is of great relevance, since it could affect the immune answer in a similar way as β-glucans derived from the cell wall components of fungi and bacteria. Typically, high-Mw β-glucans are taken up by macrophages, degraded intracellularly, and then released as short β-glucan chains extracellularly. Then, the short β-glucans stimulate macrophages and other leucocytes thereby, triggering a broad immune response [29]. 

**Figure 7:**
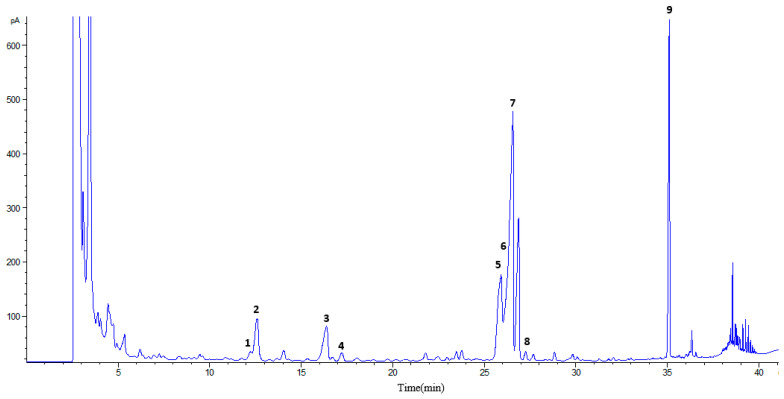
Chromatographic profile obtained by gas chromatography with flame ionization detection (GC-FID) of trimethylsilyl (TMS) oxymes of monosaccharides after hydrolysis with 2-N trifluoroacetic acid (TFA) of extracted pectin from Euglena cantabrica by PLE (110 °C with 50% of EtOH). (1) Xylose (Xyl), (2) xylose+arabinose (Xyl+Ara), (3) rhamnose (Rha), (4) fucose (Fuc), (5) galactose (Gal), (6) mannose (Man), (7) glucose (Glc), (8) galacturonic acid (Gal A), and (9) internal standard.

With the aim to gain more insight on the structure of the carbohydrates extracted from *E. cantabrica,* in the present work, we have carried out a quantification of the monomeric composition of the polymers. For this purpose, a previous hydrolysis step with trifluoroacetic acid (TFA) was necessary. The monosaccharides were derivatized and analyzed as trimethylsilyl (TMS) oximes by gas chromatography with flame ionization detection (GC-FID). Figure 7 shows the chromatographic profile of monosaccharides that constitute the carbohydrate polymers extracted by PLE from *E. cantabrica*. 

**Figure 8:**
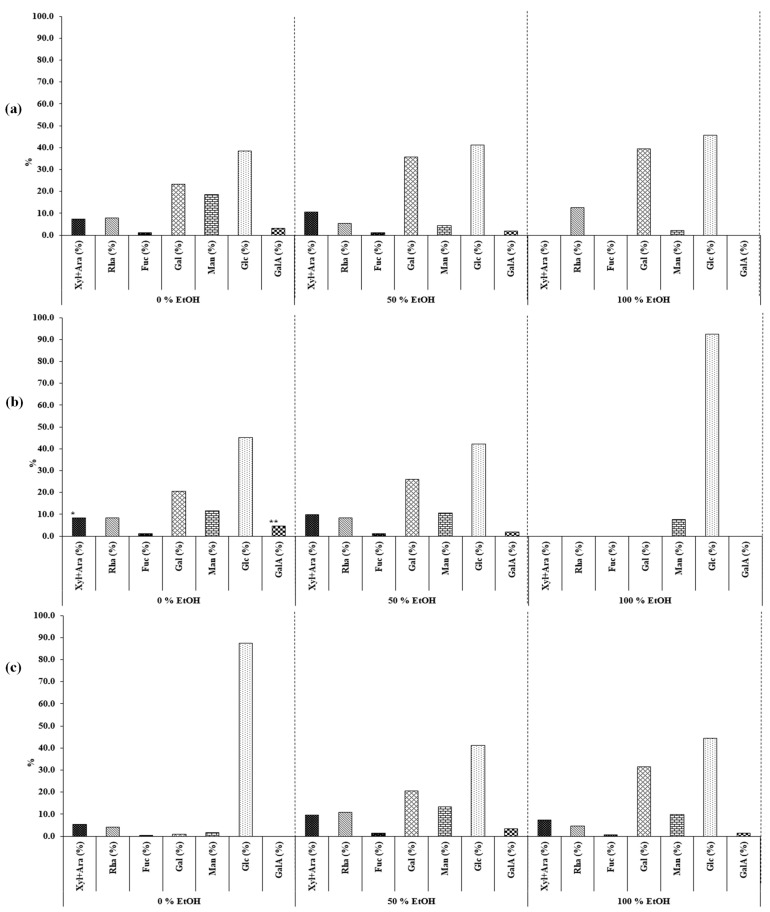
Distribution and abundance of the monomeric compositions (% over total carbohydrates) obtained by GC-FID after the hydrolysis with TFA of the extractions carried out at 40 (**a**), 110 (**b**), and 180 °C (**c**).

The most striking feature was the presence of elevated amounts of glucose, which is the unique monosaccharide present in paramylon, which, in *Euglena* spp., is a chain without branching. In addition, most of the extractions gave rise to the presence of xylose, arabinose, fucose, galactose, and mannose in variable concentrations (see Figure 8 and Table 3). O’Neill et al. [30] carried out an exhaustive study on the huge availability of *E. gracilis* for the synthesis of complex glycans and found that polymers related to xylan, mannan, arabinan, and arabinogalactan can be also present. Moreover, at the base of the flagella, some α-glucose and α-mannose residues could be at the fission point. Additionally, the N-glucan analysis of all the Euglena samples studied by these authors demonstrated that mannose is the unique monosaccharide constituting N-glycans and that the surface of *E. gracilis* has some xylan- and arabinan-type materials.

It is possible to conclude that, undoubtedly, *E. cantabrica* is a rich source of interesting carbohydrates with bioactive properties, although more studies are needed to exhaustively characterize the structure of all the glycans present. It seems clear that not a single family of compounds is responsible for the antioxidant capacity but the relative amount of carbohydrates, phenolic compounds, and pigments.

## 3. Materials and Methods

### 3.1. Strain, Culture Conditions, and Breaking of the Cell Wall

*Euglena cantabrica* (BEA 0937B) clonal strains were provided by the culture collection at the Spanish Bank of Algae (BEA) in Gran Canaria (Spain). The algae were cultivated at natural conditions with a light intensity media of 134.3 W/m^2^ and at a temperature media of 24 °C. The cultures were done with a dark:light cycle media of 12.5:11.5. Then, the cell wall of the alga was broken at 1500 bar (high-pressure homogenization). After this, the frozen paste was lyophilized before extraction. Methanol and Methyl tert-butyl ether (MTBE) were HPLC quality, from VWR (Avantor, West Chester, Pensilvania, USA)

### 3.2. Pressurized Liquid Extraction 

The extractions were done in an ASE 200 (Accelerated Solvents Extractor, Dionex, Sunnyvale, CA, USA) with the conditions established in the factorial 3^2^ DoE. Extraction temperature (40–180 °C) and solvent composition (0–100% ethanol in water) were the two experimental factors under study. The order of the experiments was randomized by the statistical software Statgraphics Centurion (version XVI) to avoid the error due to incontrollable parameters. Cross-contamination between experiments was prevented by a cleaning protocol (a blank extraction) after each extraction. All the extractions of *E. cantabrica* were done using 1 g of freeze-dried disrupted microalgae mixed with 2 g of sea sand. Extraction time was 20 min for all extractions, because it was demonstrated that the extraction time above 15 min does not have statistical influence in the yield or the antioxidant capacity [15]. Pressure was constant (10 MPa) in order to keep solvents in a liquid phase under all temperatures tested. Solvent was removed to calculate the extraction yield: ethanol was evaporated under a nitrogen stream, and water was freeze-dried.

### 3.3. Chemical Characterization and Evaluation of the Antioxidant Capacity

#### 3.3.1. Total Phenols Content (TPC): Folin-Ciocalteau Test

The total content of phenols was measured as mg of GAE (gallic acid equivalents)/g extract, according to the protocol developed by Kosar et al. [31], with some modifications [21]. Phenols existing in algae extracts were able to reduce Mo(VI) to Mo(V) present in the Folin-Ciocalteau reagent. The intensity of the blue color generated (absorbance at 760 nm) was related to the concentration of phenols in the extract. Briefly, an aliquot (10 μL) of extract solution (5 to 10 mg extract/mL) was deposited in an Eppendorf type tube together with 600 μL of ultrapure water. To this mixture, 50 μL of undiluted Folin–Ciocalteau reagent (Merck, Darmstadt, Germany) and 150 μL of 20% (w/v) Na_2_CO_3_ (1 min later) were subsequently added, and the volume was made up to 1 mL with ultrapure water. The mixture was vortexed and incubated for 2 h at room temperature in the darkness. Finally, 300 μL of each reaction mixture was transferred to a 96-well plate to measure the absorbance at 760 nm (microplate spectrophotometer reader Synergy HT, BioTek Instruments, Winooski, VT, USA). Gallic acid was used as a reference standard for calibration (0.031–2 mg mL^−1^).

#### 3.3.2. Antioxidant Capacity: ABTS Test

The ABTS (2,2’-azino-bis(3-ethylbenzothiazoline-6-sulfonic acid) diammonium salt) test was performed according to the procedure developed by Re et al. [32], with some modifications [21]. First, ABTS^•+^ radicals were generated by a reaction of ABTS (7 mM) with potassium persulfate (2.45 mM) in the dark at room temperature for 16 h. The radical solution was diluted with sodium phosphate buffer (5 mM, pH 7.4) to an absorbance value of 0.7 (± 0.02) measured at 734 nm. Then, the inhibition of the diluted radical solution by the different algae extracts (10 µL of extract mixed with 1 mL of ABTS^•+^ solution) was measured after 45 min of incubation. To this aim, five different dilutions of each extract giving a linear response between 20% and 80% of the blank absorbance were analyzed in triplicate. Results were expressed as TEAC values (mmol Trolox equivalents (TE)/g samples), since Trolox was used as the reference standard. The scavenging of the DPPH (1,1-diphenyl-2-picrylhydrazyl) radical was based on the procedure developed by Brand-Williams et al. [33], modified by the authors of [34]. Briefly, the inhibition of a solution of the radical by the different algae extracts was measured (absorbance at 516 nm) after 4 h of incubation. To do this, different dilutions of *E. cantabrica* PLE extracts (10 µL) were mixed with a solution of 60 µM DPPH (290 µL), and the absorbance after the incubation time was compared against a calibration curve of DPPH prepared in methanol (7.5–90 µM). Results are expressed as the effective concentration of extract able to decrease the concentration of DPPH to the half (EC50).

#### 3.3.3. Total Lipids

The total content of lipids was measured by weighing the dried extract obtained with methanol: chloroform (2:1), following the gravimetric protocol of Aselsson and Gentili [35].

#### 3.3.4. Carotenoids and Chlorophylls

The analysis of pigments was done using a procedure based on high-performance liquid chromatography coupled to a diode array detector and atmospheric pressure chemical ionization tandem mass spectrometry (HPLC-DAD-APCI-MS/MS). The procedure followed was a modification of the method developed by Castro-Puyana et al. [16]. Analyses of the extracts were conducted using a HPLC 1100 series (Agilent Technologies, Santa Clara, CA, USA) equipped with a diode array detector and coupled to an ion trap mass spectrometer (Esquire 2000, Bruker, Bremen, Germany) via an atmospheric pressure chemical ionization (APCI) interface. Separation of pigments was carried out in a YMC-C30 reversed-phase column (250 mm × 4.6 mm inner diameter, 5-μm particle size; YMC Europe, Schermbeck, Germany) protected by a pre-column YMC-C30 (10 mm × 4 mm i.d., 5 μm). The mobile phase was a mixture of methanol–MTBE–water (90:7:3 v/v/v) (solvent A) and methanol–MTBE (10:90 v/v) (solvent B). Mobile phase was flowing at 0.8 mL min^−1^ according to the following gradient: 0 min, 0% B; 15 min, 17% B; 20 min, 17% B; 30 min, 25% B; 35 min, 55% B; 45 min, 85% B; 50 min, 100% B; 60 min, 100% B; and 62 min, 0% B. The injection volume was 10 μL. UV-Vis spectra from 240 to 770 nm were collected using the DAD (peak width > 0.1 min (2 s), slit 4 nm), and chromatographic profiles were recorded at 450 and 660 nm. Full-scan spectrum was acquired in the range of *m/z* 150–1300. Automatic MS/MS analyses were also performed, fragmenting the two highest precursor ions (10,000-count threshold; 1-V fragmentor amplitude). Data were processed by ChemStation software from Agilent Technologies (Rev. B.04.03) and DataAnalysis from Bruker GmbH (version 3.2).

#### 3.3.5. Carbohydrates 

##### Estimation of Molecular Weight (Mw)

The Mw distributions were estimated following the method described by Muñoz-Almagro et al. (2018). Samples (0.1% w/v, 50 µL) were eluted using a TSK-Gel guard column (6.0 mm × 400 mm) and two TSK-Gel columns connected in series G5000 PWXL (7.8 mm × 300 mm, 10 μm) and G2500 PWXL (7.8 mm × 300 mm, 6 μm) (Tosoh Bioscience, Stuttgart, Germany). The mobile phase was 0.01-M NH_4_Ac as the mobile phase and pectin samples were separated at 0.5 mL/min and 30 °C during 50 min. Dextran blue was used for the establishment of the exclusion volume of the columns (1000 kDa). For the calibrations, a Pullulan Standard Set (Sigma, St. Louis, MO, USA) was used. The average molecular weight (Mw) of the different pullulans were 0.342 kDa; 1.32 kDa, 6.20 kDa, and 10 kDa; 21.7 kDa; 48.8 kDa; 113 kDa; 200 kDa; and 348 kDa and 805 kDa. 

##### Determination of Monomeric Composition 

Before pectin derivatization, a hydrolysis with TFA 2N 110 °C was carried out following the method reported by Muñoz-Almagro et al. [36]. Samples were eluted using a DB-5HT column (15 m × 0.32 mm × 0.10 μm, J&W Agilent, Folson, CA, USA) and split 1:5. The flow of nitrogen was 1 mL/min. The temperatures of the injector and detector were kept at 280 and 350 °C, respectively; the temperature program started at 150 °C and raised to 165 °C at 1 °C/min and up to 300 °C at 10 °C/min. For identification and quantitation, standard solutions of xylose, arabinose, rhamnose, fucose, galactose, mannose, glucose, and galacturonic acid over the expected concentration range in extracts of samples were injected and analyzed to calculate the response factor relative to phenyl-β-d-glucoside (internal standard, 0.05% w/v).

## 4. Conclusions

Pressurized liquid extraction using green solvents, such as water and ethanol mixtures, has demonstrated its potential to obtain different extracts of interest from *Euglena cantabrica*. Extracts with high antioxidant capacities can be obtained at 180 °C with 41.1% EtOH, according to the response surface obtained from the statistical analysis of the DoE. The presence of natural pigments such as pheophytins and the carotenoid diatoxanthin (previously reported in *E. sanguinea*) in extracts obtained with pure ethanol has been confirmed by using HPLC-DAD-MS/MS. On the other hand, the bioactive carbohydrate paramylon was more efficiently extracted at 110 °C using 50% EtOH, as it has been revealed using HPSEC-ELSD and GC-FID. This is the first study on the application of pressurized green extraction techniques to the scarcely investigated *Euglena cantabrica*, which increases the knowledge about its chemical composition and opens a huge potential of the application of this microalga for food and pharmacological uses. Biorefinery approaches based on green pressurized technologies should be further investigated, since the results of this screening show that the modulation of the extraction conditions may lead to a selective fractionation of *E. cantabrica* biomass, in order to develop a green downstream platform that yields several fractions with different industrial applications.

## Figures and Tables

**Table 1:** Results obtained for each response variable at the different extraction condition of *Euglena cantabrica* defined by the design of experiments of pressurized liquid extraction (PLE): yield, TPC, and antioxidant capacities obtained by ABTS and DPPH assays. GAE: gallic acid equivalents per gram of extract, EtOH: solvent composition, TE: Trolox equivalents, and EC_50_: effective concentration of extract (in µg/mL) able to decrease the DPPH radical concentration by 50%.

Experiment #	T, °C	%EtOH (v/v)	Extraction Yield, %(m/m)	TPC, mg GAE/g Extract	ABTS, mmol TE/g Extract	DPPH, EC_50_ (µg/mL)
Center ^(a)^	110	50	21.6 ± 1.1	45.6 ± 6.9	0.80 ± 0.02	26.8 ± 3.9
2	180	50	39.1	38.3	1.26	27.6
3	110	0	9.9	16.8	0.46	66.2
4	40	0	15.9	14.5	0.39	132.2
7	180	100	12.1	39.4	0.55	7.5
9	180	0	27.3	46.0	1.28	29.7
10	110	100	11.6	30.1	0.32	33.2
11	40	100	3.2	19.5	0.22	67.8
12	40	50	12.9	34.3	0.73	30.0

^(a)^Average results (± SD) from the four replicates of the center points of the design of experiments (DoE) corresponding to the experiments 1, 5, 6, and 8.

**Table 2:** The identification of pigments in a *Euglena cantabrica* PLE extract (110 °C, 100% ethanol) by HPLC-DAD-APCI-MS/MS. sh: shoulder.

Peak #	Rt (min)	Identification	Absorbance Max (nm)	Parent *m/z*	Fragments *m/z*
1	2.2	Carotenoid	272, 424(sh), 448, 474		
2	5.3	Chlorophyll	404, 660		
3	5.8	Chlorophyll	402, 662		
4	6.6	Chlorophyll	408, 660		
5	8.8	Chlorophyll	438, 654		
6	9.5	Carotenoid	402(sh), 422, 446	583.6	491.6
7	11.0	Pheophorbide *a*	408, 666	593.5	533.5, 451.5
8	12.5	Carotenoid	426(sh), 448		
9	12.8	Carotenoid	426(sh), 448		
10	13.2	Carotenoid	422, 446(sh)	594.6	494.4
11	13.8	Carotenoid	450, 472(sh)		
12	14.8	Pyropheophorbide b	438, 654	549.4	521.5
13	15.6	Carotenoid	444, 472		
14	15.9	Carotenoid	446, 468		
15	16.9	Carotenoid	408(sh), 430, 456	506.9	268.4
16	18.0	Pyropheophorbide *a*	412, 666	535.5	
17	18.7	Not identified			
18	19.6	Diatoxanthin	450, 478	567.5	549.7
19	20.5	Chlorophyll	418, 660	889.7	611.3, 551.5
20	21.8	Carotenoid	426(sh), 450, 478	557.7	291.4
21	23.1	Chlorophyll	418, 660	959.8	
22	25.4	Hydroxypheophytin b	438, 472(sh), 654	901.6	873.8, 623.5
23	27.4	Chlorophyll	418, 664	887.6	869.6, 609.5
24	29.5	Hydroxypheophytin *a*	404, 666	887.8	869.8, 609.5
25	31.7	Chlorophyll	416, 664	852.1, 887.8	
26	32.6	Chlorophyll	434, 660	823.8	805.8, 567.5
27	33.5	Pheophytin b and b’	436, 654	885.8	607.4, 547.3
28	35.2		436, 654	885.8	607.5, 547.4
29	36.7	Pheophytin *a* and *a’*	408, 666	871.9	593.5, 533.5
30	37.2		408, 666	871.9	593.5, 533.5
31	38.3	Chlorophyll	418, 664	871.9	856
32	38.6	Carotenoid	426(sh), 450, 478		
33	39.2	Carotenoid	420(sh), 444, 472	603.6	265.3
34	39.6	Chlorophyll	418, 664	955.9	676.6, 616.6
35	40.2	Pyropheophytin b	436, 654	827.5	549.5
36	40.9	Not identified	466, 492(sh), 662		
37	41.1	Pyropheophytin *a* and *a’*	410, 668	813.9	535.5
38	41.2		410, 668	813.9	535.5
39	42.3	Chlorophyll	420, 664		
40	43.9	Carotenoid	438(sh), 470, 502	857.9	591.5, 441.6
41	49.4	Carotenoid	468(sh), 494, 528		
42	51.7	Not identified		517.5	499.4, 488.5

**Table 3:** Distribution of the molecular weights and abundance of the monomeric composition (% over total carbohydrates) obtained by gas chromatography with flame ionization detection (GC-FID) after the hydrolysis with trifluoroacetic acid (TFA) of the extractions was carried out at different temperatures and solvent compositions.

**Temp.**	**Solvent**	**Molecular weights of the carbohydrate fractions**						
		>805 kDa	804–89 kDa	89–24 kDa	24–5 kDa	5–1 kDa	1-0.4 kDa	0.39–<0.342 kDa
40 ˚C	0% EtOH	>805 (18%)	696 (14%)	52.5 (8%)	8.4 (8%)	3.8 (19%)	0.5 (4%)	<0.342 (29%)
	50% EtOH	>805 (4%)	483.1 (8%)	24.3 (5%)	7.5 (12%)	3.7 (24%)	0.9 (9%)	<0.342 (38%)
	100% EtOH	>805 (21%)	-	-	-	2 (24%)	-	<0.342 (55%)
110 ˚C	0% EtOH	>805 (9%)	651.3 (32%)	-	8.1 (17%)	3.8 (25%)	0.4 (8%)	<0.342 (7%)
	50% EtOH	>805 (15%)	505.6 (11%)	22.8 (9%)	8.2 (12%)	3.8 (16%)	0.8 (12%)	<0.342 (25%)
	100% EtOH	>805 (49%)	-	-	-	4.5 (19%)	0.6 (32%)	-
180 ˚C	0% EtOH	>805 (11%)	-	26.1 (10%)	8.1 (35%)	3.2 (35%)	-	<0.342 (9%)
	50% EtOH	>805 (14%)	585.2 (5%)	17.3 (16%)	8.1 (19%)	3.2 (30%)	-	<0.342 (16%)
	100% EtOH	>805 (36%)	-	-	5.2 (9%)	3 (21%)	0.5 (12%)	<0.342 (22%)
